# Immunogenicity, Reactogenicity, and Safety of a Pentavalent Meningococcal ABCWY Vaccine in Adolescents and Young Adults Who Had Previously Received a Meningococcal ACWY Vaccine: A Phase 3, Randomized Controlled Clinical Study

**DOI:** 10.1093/cid/ciae622

**Published:** 2024-12-26

**Authors:** Terry Nolan, Chiranjiwi Bhusal, Alejandro Hoberman, Conrado J Llapur, Olga Voloshyna, Ezekiel Fink, Angela Gentile, Garry Wallace, Peter C Richmond, Joseph B Domachowske, Thembile Mzolo, Maria Lattanzi, Daniela Toneatto, for the BOOST Study Group, Adebayo Akinsola, Madhavi Ampajwala, Mark Arya, Andrew Bartlett, Divya Batra, Mark Theo Bloch, Jose Bordon, William Byars, Jeremy Peter James Carr, Ana Ceballos, Ferdinandus de Looze, Mercedes Deluca, Joseph Domachowske, Rand Farjo, Ezekiel Fink, Angela Gentile, Jennifer Gilsoul, Elizabeth Anne Gunner, Anil K Gupta, Alejandro Hoberman, Matthew Hong, Julie Kasarjian, Conrado Juan Llapur, Darvy Mann, Gonzalo Perez Marc, Paul G Matherne, Gretchen Mitchell, Terry Nolan, Mora Nair Obed, John OħMahony, Peter Richmond, Fernando Oscar Riera, Walter Rok, Louis Saravolatz, Peter Silas, Adriana Elvira Soto, Katherine Sullivan, Joseph Surber, Ricardo Augusto Teijeiro, Florence Tiong, Olga Voloshyna, Ushma Wadia, Garry Wallace, Clifford Yut

**Affiliations:** Peter Doherty Institute at the University of Melbourne, VIC, Australia; Murdoch Children's Research Institute, Melbourne, VIC, Australia; GSK, Amsterdam, The Netherlands; University of Pittsburgh School of Medicine, Pittsburgh, Pennsylvania; Hospital del Niño Jesús, San Miguel de Tucumán, Argentina; Northside Health, Coffs Harbour, New South Wales, Australia; Cedar Health Research, LLC, Dallas, Texas; Hospital de Niños Dr Ricardo Gutierrez, Buenos Aires, Argentina; Dawson Clinical Research, Guelph, Ontario, Canada; University of Western Australia, Perth, Western Australia, Australia; Perth Children’s Hospital, Perth, Western Australia, Australia; Vaccine Trials Group, Telethon Kids Institute, Nedlands, Western Australia, Australia; State University of New York Upstate Medical University, Syracuse, New York; GSK, Amsterdam, The Netherlands; GSK, Siena, Italy; GSK, Siena, Italy

**Keywords:** immunogenicity, MenABCWY, MenACWY, primed, safety

## Abstract

**Background:**

A MenABCWY vaccine containing 4CMenB and MenACWY-CRM vaccine components has been developed to protect against the 5 meningococcal serogroups that cause most invasive disease cases.

**Methods:**

In this phase 3 study, healthy participants aged 15–25 years, who had received MenACWY vaccination ≥4 years previously, were randomized (1:1) to receive 2 MenABCWY doses 6 months apart or 1 MenACWY-CRM dose. Primary objectives were to demonstrate the noninferiority of MenABCWY 1 month postvaccination versus MenACWY-CRM, with a lower limit of 2-sided 95% confidence interval above −10% for group differences in 4-fold rise in human serum bactericidal antibody (hSBA) titers against serogroups ACWY, and to evaluate reactogenicity and safety. Secondary endpoints included percentages of participants with hSBA titers greater than or equal to the lower limit of quantitation (≥LLOQ) against serogroups ACWY and vaccine antigen-specific serogroup B (MenB) indicator strains.

**Results:**

Noninferiority of MenABCWY versus MenACWY-CRM was demonstrated following each MenABCWY dose. Percentages of participants with hSBA titers ≥LLOQ for serogroups ACWY were 97.9%–98.9% and 99.5%–100% following 1 and 2 MenABCWY doses, respectively, and 96.8%–99.0% following 1 MenACWY-CRM dose. After 2 MenABCWY doses, 75.6%–96.3% of participants had hSBA titers ≥LLOQ against MenB indicator strains. The MenABCWY vaccine was well tolerated in MenACWY-primed individuals, with a favorable safety profile.

**Conclusions:**

Immune responses against serogroups ACWY following 1 and 2 doses of investigational MenABCWY vaccine are noninferior to those following MenACWY-CRM in MenACWY-primed adolescents and young adults. Robust immune responses were observed against MenB indicator strains after 2 MenABCWY doses administered 6 months apart.

**Clinical Trials Registration.** NCT04707391.

## Teaser

Immune responses against serogroups A, C, W, and Y following 1 and 2 doses of MenABCWY candidate vaccine are noninferior to those following MenACWY-CRM in MenACWY-primed adolescents and young adults. MenABCWY vaccine had a favorable safety profile.

## Graphical Abstract

**Graphical Abstract:**
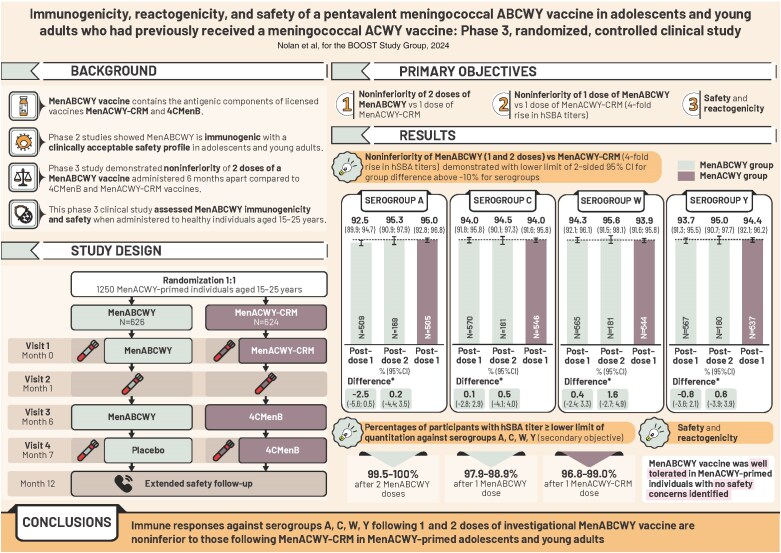

Invasive meningococcal disease (IMD) is a life-threatening disease that most often presents as meningitis or sepsis but can have nonspecific clinical features initially [1, 2]. The incidences of IMD are highest in young children, adolescents and young adults, and older adults [3, 4]. Vaccines are available against the 5 serogroups that cause most IMD cases [5]: quadrivalent vaccines that target the polysaccharide capsule of meningococcal serogroups A, C, W, and Y (MenACWY) and monovalent protein-based vaccines against meningococcal serogroup B (MenB). Additionally, a pentavalent MenABCWY vaccine (Penbraya, Pfizer) was licensed recently [6] and GSK's MenABCWY vaccine [7] is under review by the United States (US) Food and Drug Administration.

In the US, MenACWY vaccination is recommended at age 11 or 12 years with a booster dose 5 years later because of antibody waning [8]. This booster dose is critical for maintaining protection during late adolescence and early adulthood [9], but booster dose coverage is suboptimal, with only 61% of 17-year-olds vaccinated with 2 or more doses in 2022 [10]. In most other countries, at least 1 dose is recommended between the ages of 11 and 25 years [11]. Few countries routinely vaccinate adolescents and young adults against MenB despite the increased risk of MenB disease in that age group [11]. MenB vaccination (or vaccination with licensed MenABCWY vaccine when MenACWY and MenB are indicated at the same visit) is recommended in the US for individuals aged 16–23 years based on shared clinical decision making [8, 12]. In Europe and other regions, the 4-component MenB vaccine, 4CMenB (Bexsero, GSK), is licensed for those aged 2 months or older [13] and bivalent MenB-FHbp vaccine (Trumenba, Pfizer) for individuals from 10 years old [14]. 4CMenB is included in various national/regional childhood immunization programs in Europe, South America, and Oceania, with several also including 4CMenB in the adolescent immunization program [15].

GSK's MenABCWY vaccine is a combination of MenACWY CRM_197_-glycoconjugate vaccine (MenACWY-CRM; Menveo, GSK) [16] and 4CMenB, containing antigenic components *Neisseria* adhesin A (NadA), neisserial heparin-binding antigen (NHBA), factor H binding protein (fHbp), and outer membrane vesicle proteins, including Porin A (PorA) [7, 13, 17]. The safety and effectiveness of MenACWY-CRM and 4CMenB are confirmed by real-world data from >10 years of use [15, 18]. Phase 2 studies of the MenABCWY vaccine demonstrated its immunogenicity and clinically acceptable safety profile in adolescents and young adults [19–26]. A phase 3 study in healthy individuals aged 10–25 years demonstrated noninferiority versus the licensed parent vaccines and breadth of immune response against a diverse invasive 110 MenB strain panel [27].

The present phase 3 clinical study was conducted in healthy adolescents and young adults primed with a MenACWY vaccine, with the primary objective of demonstrating the noninferiority of immune responses induced by MenABCWY, when administered as a booster (for the MenACWY component), to those induced by MenACWY-CRM. The reactogenicity and safety of both vaccines was also assessed.

## METHODS

### Study Design and Participants

This phase 3, randomized, controlled, observer-blind study (ClinicalTrials.gov identifier NCT04707391) was conducted between January 2021 and May 2023 at 65 centers in 4 countries (Argentina, Australia, Canada, and US) in accordance with the Declaration of Helsinki and Good Clinical Practice, and with the approval of appropriate ethics committees. Written informed consent, and assent for participants aged <18 years, was provided before enrollment by participants or their parents or legally acceptable representatives.

Healthy adolescents and young adults aged 15–25 years with no history of meningococcal disease, who had received a single dose of any licensed MenACWY vaccine ≥4 years before enrollment, were recruited. Previous vaccination with >1 MenACWY dose or with a MenB vaccine was not allowed. All inclusion/exclusion criteria for participation are listed in the study protocol (available at https://www.gsk-studyregister.com/en/; study identifier: 213171).

Participants were randomized (1:1 ratio) to receive 2 doses of the investigational MenABCWY vaccine at 0 and 6 months (MenABCWY group) or 1 MenACWY-CRM dose at month 0 (MenACWY group). Two doses of 4CMenB were administered to the MenACWY group at months 6 and 7. The MenABCWY group received a placebo injection (0.9% saline solution) at month 7. Vaccine preparation and injection methods are described in the Supplementary Material.

Allocation to study group at each site was conducted via a central randomization system using a minimization procedure accounting for country. There were 4 study visits and regular safety follow-up through telephone calls to each participant, with the final call 12 months after the first vaccine dose.

The study was observer blinded; that is, participants, investigators, and teams responsible for assessing study endpoints were unaware of which vaccine was administered, and vaccines were prepared and administered by qualified healthcare professionals who did not participate in data management or review. The study laboratory was blinded to treatment, subject, and visit number.

Primary study objectives were to demonstrate the noninferiority of immune responses against serogroups A, C, W, and Y 1 month after the first and the second MenABCWY dose compared to 1 dose of MenACWY-CRM, and to evaluate the safety and reactogenicity of both vaccines. Other immunogenicity endpoints against serogroups A, C, W, and Y, and against the MenB component of the MenABCWY vaccine, were evaluated as secondary objectives. All primary and secondary study endpoints are described in the Supplementary Material.

### Serological Analyses

Blood samples were collected at the first visit (baseline) and postvaccination at months 1 and 7. Immunogenicity of the MenACWY component of MenABCWY and MenACWY-CRM was evaluated by human serum bactericidal antibody (hSBA) assay against serogroups A, C, W, and Y. Immunogenicity of the MenB component of MenABCWY vaccine was assessed by hSBA assay against 4 indicator strains, each expressing 1 of the 4 vaccine antigens (fHbp, NadA, NHBA, or PorA) on its surface [28]. Immunogenicity endpoints included percentages of participants with 4-fold rises in hSBA titer and hSBA titers greater than or equal to the lower limit of quantitation (≥LLOQ; see Supplementary Material), and hSBA geometric mean titers (GMTs).

### Reactogenicity and Safety

Participants were observed for ≥30 minutes after each vaccine dose for immediate adverse events (AEs). Solicited administration site (injection site pain, erythema, swelling, induration) and systemic (fever, nausea, fatigue, myalgia, arthralgia, headache) AEs were reported by participants on electronic diaries for 7 days following vaccination at months 0 and 6. Solicited AEs were classified as mild, moderate, or severe, with severe defined as preventing normal activity, apart from severe erythema, swelling, and induration, which were defined as diameter >100 mm. Fever was defined as body temperature ≥38.0°C and severe fever as body temperature ≥40.0°C.

Unsolicited AEs were recorded during the 30-day periods after vaccination at months 0 and 6. Serious AEs (SAEs), medically attended AEs, AEs leading to withdrawal, and AEs of special interest (AESIs) [29] were recorded over the 12-month study period. Causal relationship to vaccination was assessed by study investigators.

### Statistical Analysis

The sample size was based on results from a phase 2 MenABCWY booster study [24] (see Supplementary Material and study protocol). Estimating a 10% dropout, planned enrollment was 270 participants per group to provide 99% power and demonstrate noninferior immune responses against serogroups A, C, W, and Y following 2 MenABCWY doses versus 1 MenACWY-CRM dose, and 603 participants per group to provide 91% power and demonstrate noninferiority of 1 MenABCWY dose versus 1 MenACWY-CRM dose. Participants who withdrew or were lost to follow-up, and missing/nonevaluable measurements, were not replaced.

The 2 primary immunogenicity objectives were tested sequentially, with full alpha propagation, in the per-protocol set (participants who received ≥1 vaccine dose and had postvaccination immunogenicity data and no major protocol deviations). Noninferiority was demonstrated at each step if the lower limit of the 2-sided 95% confidence interval (CI) for the difference between the MenABCWY group and MenACWY group in percentages of participants with the seroresponse of 4-fold rise in hSBA titers 1 month postvaccination was above −10% for each serogroup (A, C, W, and Y). If noninferiority was demonstrated with the second MenABCWY dose, the second primary immunogenicity objective was tested, that is, demonstration of noninferiority after 1 MenABCWY dose versus the single MenACWY-CRM dose.

All participants who received study vaccination (dose 1 and dose 2) and provided safety data were included in the descriptive safety analyses. For the secondary immunogenicity objectives, the full analysis set (participants who received ≥1 vaccine dose and had postvaccination immunogenicity data) was analyzed, with 2-sided 95% Clopper-Pearson CIs [30].

Statistical analyses were performed using SAS version 9.4 software (SAS Institute Inc).

## RESULTS

### Participants

**Figure 1.**
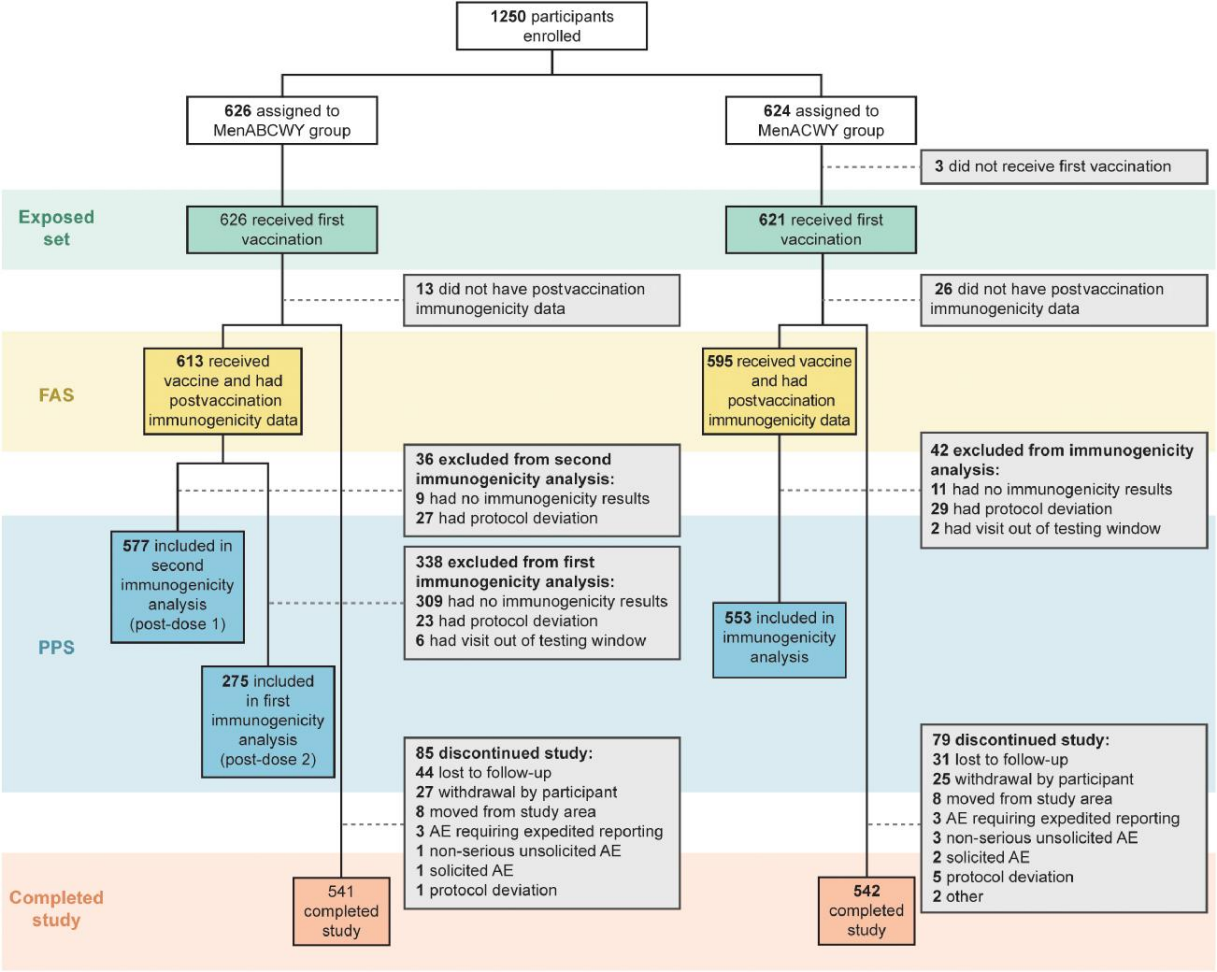
Profile of study participants randomized to the MenABCWY and MenACWY groups. Abbreviations: AE, adverse event; FAS, full analysis set; MenABCWY group, received meningococcal serogroups ABCWY vaccine at each injection; MenACWY group, received meningococcal serogroups ACWY CRM_197_-glycoconjugate vaccine at injection 1, 4CMenB at injection 2; PPS, per-protocol set.

A total of 1250 participants were enrolled and randomly allocated to receive MenABCWY (n = 626) or MenACWY-CRM (n = 624) (Figure 1). Of 1247 participants who received ≥1 study vaccine dose, 1083 completed the study; 164 (13.2%) withdrew prematurely, most frequently because of loss to follow-up (75 participants [6.0%]) and withdrawal by participant (52 participants [4.2%]) (Figure 1). A total of 1208 participants were included in the full analysis set and, in the per-protocol set, 275 participants in the MenABCWY group were included in the first primary immunogenicity analysis (post–dose 2) and 577 in the second primary immunogenicity analysis (post–dose 1), and 553 were included in the MenACWY group immunogenicity analyses (Figure 1).

The baseline characteristics of randomized participants were balanced between groups (Table 1). Overall, 731 participants (58.5%) were from the US, 238 (19.0%) from Australia, 232 (18.6%) from Argentina, and 49 (3.9%) from Canada.

**Table 1.** Demographic Characteristics of Enrolled Participants Randomized to the MenABCWY Group and MenACWY Group

Characteristic	MenABCWY Group (n = 626)	MenACWY Group (n = 624)
Age, y, mean (SD)	17.2 (2.5)	17.2 (2.6)
Age group, No. (%)		
15–17 y	450 (71.9)	441 (70.7)
18–25 y	176 (28.1)	180 (28.8)
Sex, No. (%)		
Male	283 (45.2)	299 (47.9)
Female	343 (54.8)	325 (52.1)
Race, No. (%)		
White	474 (75.7)	467 (74.8)
Black or African American	94 (15.0)	86 (13.8)
Asian	22 (3.5)	33 (5.3)
Other	36 (5.8)	38 (6.1)

Abbreviations: MenABCWY group, received 2 doses of meningococcal serogroups ABCWY vaccine at study months 0 and 6; MenACWY group, received meningococcal serogroups ACWY CRM_197_-glycoconjugate vaccine at month 0; SD, standard deviation; y, years.

### Immunogenicity

Noninferiority of 2 MenABCWY doses administered 6 months apart versus the single MenACWY-CRM dose was demonstrated by hSBA assay, with lower limits of 2-sided 95% CIs for the group differences in 4-fold rise in hSBA titers above the predefined criterion, −10%, against serogroups A, C, W, and Y (Table 2). Noninferiority of 1 MenABCWY dose was also demonstrated (Table 2).

**Table 2.** Percentages of Participants With 4-Fold Rise From Baseline in Human Serum Bactericidal Antibody Titers Against Meningococcal Serogroups A, C, W, and Y, 1 Month After 1 (Month 1) or 2 (Month 7) MenABCWY Doses and 1 Month After 1 MenACWY-CRM Dose (Month 1) (Per-Protocol Set)

Serogroup Timepoint	Percentages of Participants With 4-Fold Rise in hSBA Titers^a^				Difference^b^
	MenABCWY Group		MenACWY Group		
	No. of Participants	% (95% CI)	No. of Participants	% (95% CI)	% (95% CI)
Serogroup A					
Month 1	509	92.5 (89.9–94.7)	505	95.0 (92.8–96.8)	−2.5 (−5.6 to .5)
Month 7	169	95.3 (90.9–97.9)	…	…	0.2 (−4.4 to 3.5)
Serogroup C					
Month 1	570	94.0 (91.8–95.8)	546	94.0 (91.6–95.8)	0.1 (−2.8 to 2.9)
Month 7	181	94.5 (90.1–97.3)	…	…	0.5 (−4.1 to 4.0)
Serogroup W					
Month 1	565	94.3 (92.1–96.1)	544	93.9 (91.6–95.8)	0.4 (−2.4 to 3.3)
Month 7	181	95.6 (91.5–98.1)	…	…	1.6 (−2.7 to 4.9)
Serogroup Y					
Month 1	567	93.7 (91.3–95.5)	537	94.4 (92.1–96.2)	−0.8 (−3.6 to 2.1)
Month 7	180	95.0 (90.7–97.7)	…	…	0.6 (−3.9 to 3.9)

Abbreviations: CI, confidence interval; hSBA, human serum bactericidal antibody; MenABCWY group, received 2 doses of meningococcal serogroups ABCWY vaccine at study months 0 and 6; MenACWY group, received meningococcal serogroups ACWY CRM_197_-glycoconjugate vaccine at month 0.

^a^Defined as postvaccination hSBA titer ≥16 when prevaccination hSBA titer <4; postvaccination hSBA titer ≥4 times the lower limit of quantitation (LLOQ) when prevaccination hSBA titer ≥limit of detection (LOD) but <LLOQ; postvaccination hSBA titer ≥4 times prevaccination titer when prevaccination hSBA titer ≥LLOQ. LOD 4 for serogroups A, C, W, and Y; LLOQ 12, 8, 8, and 10, respectively. Minor changes to the limits requested by the Center for Biologics Evaluation and Research (US Food and Drug Administration) had no clinically relevant impact on the results.

^b^Difference between MenABCWY group (month 1 or 7) and MenACWY group (month 1).

Baseline percentages of participants with hSBA titers ≥LLOQ were lowest for serogroup A (27.7% in MenABCWY group, 28.8% in MenACWY group) and highest for serogroup C (57.7% and 56.2%, respectively) (Table 3). One month postvaccination, percentages for serogroups A, C, W, and Y were 99.5%–100% following 2 MenABCWY doses, 97.9%–98.9% following 1 MenABCWY dose, and 96.8%–99.0% following 1 MenACWY-CRM dose (Table 3).

**Table 3.** Percentages of Participants at Each Timepoint With Human Serum Bactericidal Antibody Titers Against Serogroups A, C, W, and Y Greater Than or Equal to the Lower Limit of Quantitation at Baseline and 1 Month After 1 (Month 1) and 2 (Month 7) MenABCWY Doses and 1 Month (Month 1) and 6 Months (Month 7) After the Single MenACWY-CRM Dose (Full Analysis Set)

Serogroup Timepoint	Percentage of Participants With hSBA Titers ≥LLOQ^a^			
	MenABCWY Group		MenACWY Group	
	No. of Participants	% (95% CI)	No. of Participants	% (95% CI)
Serogroup A				
Baseline	546	27.7 (23.9–31.6)	549	28.8 (25.0–32.8)
Month 1	605	98.3 (97.0–99.2)	585	98.1 (96.7–99.1)
Month 7	213	99.5 (97.4–100)	585	98.1 (96.7–99.1)
Serogroup C				
Baseline	601	57.7 (53.7–61.7)	584	56.2 (52.0–60.2)
Month 1	609	98.9 (97.7–99.5)	593	99.0 (97.8–99.6)
Month 7	211	100 (98.3–100)	593	99.0 (97.8–99.6)
Serogroup W				
Baseline	597	36.3 (32.5–40.4)	583	33.4 (29.6–37.4)
Month 1	607	98.4 (97.0–99.2)	592	96.8 (95.0–98.1)
Month 7	212	100 (98.3–100)	592	96.8 (95.0–98.1)
Serogroup Y				
Baseline	600	37.5 (33.6–41.5)	576	34.9 (31.0–38.9)
Month 1	606	97.9 (96.4–98.9)	591	97.6 (96.1–98.7)
Month 7	210	100 (98.3–100)	591	97.6 (96.1–98.7)

Abbreviations: CI, confidence interval; hSBA, human serum bactericidal antibody; LLOQ, lower limit of quantitation; MenABCWY group, received 2 doses of meningococcal serogroups ABCWY vaccine at study months 0 and 6; MenACWY group, received meningococcal serogroups ACWY CRM_197_-glycoconjugate vaccine at month 0.

^a^LLOQ for serogroups A, C, W, and Y: 12, 8, 8, and 10, respectively. Minor changes to the limits requested by the Center for Biologics Evaluation and Research (US Food and Drug Administration) had no clinically relevant impact on the results.

For the MenB component of the MenABCWY vaccine, percentages of participants with hSBA titers ≥LLOQ against fHbp, NadA, NHBA, and PorA indicator strains were, at baseline, 8.7%, 7.7%, 20.2%, and 2.7%, respectively, and after 2 MenABCWY doses, 88.5%, 95.8%, 96.3%, and 75.6%, respectively (Table 4). Percentages of participants with 4-fold rises in hSBA titers were 11.6%, 49.7%, 20.4%, and 14.4%, respectively, after 1 MenABCWY dose and 68.1%, 90.1%, 64.6%, and 45.7%, respectively, after 2 doses (Table 4). Postvaccination antibody GMTs and baseline/postvaccination GMT ratios showed that robust immune responses were induced by each vaccine dose against serogroups A, C, W, and Y (Supplementary Table 1) and by 2 MenABCWY doses against each MenB indicator strain (Supplementary Table 2).

**Table 4.** Percentages of Participants in the MenABCWY Group With Human Serum Bactericidal Antibody (hSBA) Titers Greater Than or Equal to the Lower Limit of Quantitation and With 4-Fold Rise From Baseline in hSBA Titers Against Each Meningococcal Serogroup B Indicator Strain (Full Analysis Set)

MenB Indicator Strain Timepoint	hSBA Titers ≥LLOQ^a^		4-Fold Rise in hSBA Titers^b^	
	No. of Participants	% (95% CI)	No. of Participants	% (95% CI)
fHbp				
Baseline	184	8.7 (5.1–13.7)	…	…
Month 1	183	37.7 (30.7–45.2)	181	11.6 (7.3–17.2)
Month 7	165	88.5 (82.6–92.9)	163	68.1 (60.4–75.2)
NadA				
Baseline	183	7.7 (4.3–12.5)	…	…
Month 1	184	66.8 (59.5–73.6)	181	49.7 (42.2–57.2)
Month 7	165	95.8 (91.5–98.3)	162	90.1 (84.5–94.3)
NHBA				
Baseline	183	20.2 (14.7–26.8)	…	…
Month 1	184	50.0 (42.6–57.4)	181	20.4 (14.8–27.1)
Month 7	164	96.3 (92.2–98.7)	161	64.6 (56.7–72.0)
PorA				
Baseline	184	2.7 (.9–6.2)	…	…
Month 1	183	24.6 (18.5–31.5)	181	14.4 (9.6–20.3)
Month 7	164	75.6 (68.3–82.0)	162	45.7 (37.8–53.7)

Abbreviations: CI, confidence interval; fHbp, factor H binding protein; hSBA, human serum bactericidal antibody; LLOQ, lower limit of quantitation; MenABCWY group, received 2 doses of meningococcal serogroups ABCWY vaccine at study months 0 and 6; MenB, meningococcal serogroup B; NadA, *Neisseria* adhesin A; NHBA, neisserial heparin-binding antigen; PorA, Porin A.

^a^LLOQ of 5, 15, 4, and 6 for fHbp, NadA, NHBA, and PorA, respectively.

^b^Defined as postvaccination hSBA titer ≥16 when prevaccination hSBA titer <4; postvaccination hSBA titer ≥4 times LLOQ when prevaccination hSBA titer ≥limit of detection (LOD) but <LLOQ; postvaccination hSBA titer ≥4 times prevaccination titer when prevaccination hSBA titer ≥LLOQ. LOD 3 for fHbp, 6 for NadA, and 4 for NHBA and PorA. Minor changes to the limits requested by the Center for Biologics Evaluation and Research (US Food and Drug Administration) had no clinically relevant impact on the results.

### Safety

In the MenABCWY group, solicited AEs were reported by 84.5% (95% CI: 81.4%–87.3%) of participants after the first dose and 70.9% (95% CI: 67.0%–74.6%) after the second dose. In the MenACWY group, solicited AEs were reported by 59.9% (95% CI: 55.9%–63.8%) of participants after MenACWY-CRM at month 0 and 76.7% (95% CI: 73.0%–80.1%) after 4CMenB at month 6. Overall percentages of participants reporting solicited administration site or systemic AEs were similar between groups after each vaccine dose, apart from the percentage reporting administration site AEs after the first dose, which was lower following MenACWY-CRM (31.7% [95% CI: 28.1%–35.5%]) than following MenABCWY (78.0% [95% CI: 74.5%–81.1%]) (Supplementary Table 3).

**Figure 2.**
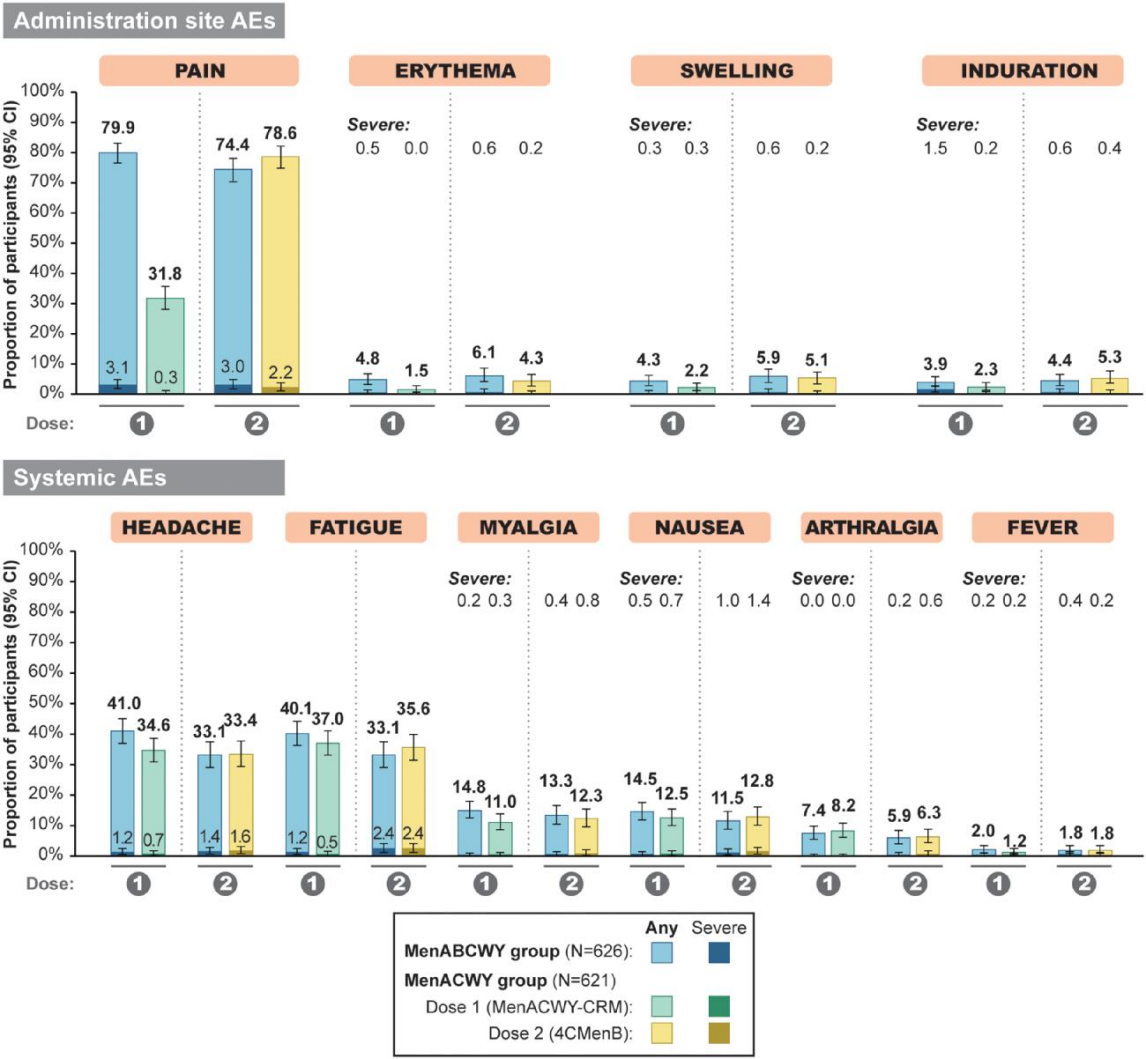
Percentages of participants reporting solicited administration site and systemic adverse events within 7 days of vaccination (solicited safety set). Severe defined as preventing normal activity; or, for erythema, swelling, and induration, diameter >100 mm; or, for fever, body temperature ≥40.0°C. Abbreviations: AEs, adverse events; CI, confidence interval; MenABCWY group, received meningococcal serogroups ABCWY vaccine at each injection; MenACWY group, received meningococcal serogroups ACWY CRM_197_-glycoconjugate vaccine at injection 1, 4CMenB at injection 2; N, number of participants in group.

After each MenABCWY dose and after MenACWY-CRM (MenACWY group, dose 1 only), injection site pain was the most commonly reported solicited administration site AE, while fatigue and headache were the most commonly reported solicited systemic AEs (Figure 2). Most solicited AEs were mild to moderate in intensity, with severe administration site events reported in ≤1.5% of participants across study groups, apart from severe pain at the MenABCWY injection site, which was reported by 3.1% and 3.0% of participants after the first and second MenABCWY dose, respectively (Figure 2). Severe systemic AEs were reported by ≤2.4% of participants after each MenABCWY dose and MenACWY-CRM (Figure 2). Severe fever was reported by 1 participant in each group after the first dose, 2 participants in the MenABCWY group after the second dose, and 1 participant in the MenACWY group after 4CMenB administration. The mean duration of solicited AEs was <4 days (data not shown) and no increase in reporting frequency was observed following the second MenABCWY dose (Figure 2).

During the 30-day follow-up period after vaccination, 24.0% of participants in the MenABCWY group and 24.2% in the MenACWY group reported ≥1 unsolicited AEs (Supplementary Table 4). During the entire study, AEs considered by the study investigators to be at least possibly related to vaccination were reported by no more than 2 participants in each group, apart from injection site pain, which was reported by 4 participants in the MenACWY group after 4CMenB administration.

During the study, SAEs were reported in 18 participants (2.9%) in the MenABCWY group and 7 (1.1%) in the MenACWY group, none of which were assessed by the investigator as causally related to vaccination (Supplementary Table 4). Percentages of participants reporting a medically attended unsolicited AE were 35.6% in the MenABCWY group and 33.2% in the MenACWY group (Supplementary Table 4). The most frequently reported medically attended AEs were coronavirus disease 2019 (2.6% and 2.9%, respectively), influenza (1.8% and 1.0%), upper respiratory tract infection (1.9% and 0.8%), urinary tract infection (1.6% and 1.1%), and anxiety (2.1% and 1.3%). There was 1 death in each group, both due to suicide and assessed as not causally related to vaccination. Six participants were reported to have unsolicited AEs leading to discontinuation or delay in study vaccination (2 participants in MenABCWY group, 4 in MenACWY group), 1 of which (diarrhea in MenACWY group) was assessed as causally related to vaccination by the investigator. Four AESIs (celiac disease, ulcerative colitis, Crohn disease, and arthritis) were reported in 1 participant each in the MenACWY group. All were preexisting chronic conditions, apart from ulcerative colitis, which was newly diagnosed 20 days after the participant received 4CMenB. All AESIs were assessed as nonserious and not related to study intervention.

## DISCUSSION

This phase 3 clinical study assessed the immunogenicity and safety of a pentavalent MenABCWY vaccine that contains the antigenic components of the licensed 4CMenB and MenACWY-CRM vaccines when administered in a 2-dose schedule (0–6 months) to healthy adolescents and young adults who had received a MenACWY vaccine dose ≥4 years previously.

A booster dose of quadrivalent conjugate vaccine is recommended for adolescents in various countries to ensure protection in an age group at higher risk of IMD [8, 11]. In prior MenACWY-CRM studies, a booster dose administered 3–6 years after primary MenACWY vaccination induced a robust anamnestic response [18]. Similarly, in our study, administration of quadrivalent or pentavalent meningococcal vaccine as booster dose resulted in substantial increases in antibody titers against serogroups A, C, W, and Y. Additionally, 1 and 2 doses of MenABCWY were both noninferior to the single MenACWY-CRM dose, as assessed by the percentages of participants with 4-fold rises in hSBA titers from baseline. The MenB component of the MenABCWY vaccine was also demonstrated to be immunogenic in this group of participants, who had not received a MenB vaccine previously, with robust immune responses induced against each MenB indicator strain following 2 MenABCWY doses.

Observed anamnestic responses against serogroups A, C, W, and Y were consistent with those seen in previous studies of MenABCWY booster doses administered 24 or 48 months after primary vaccination with MenABCWY or MenACWY-CRM [21, 23, 24]. The immune responses against the 4 MenB indicator strains following 2 doses of the MenABCWY vaccine are also in line with previous reports [19, 20, 22, 23, 25, 26, 31]. In the prior phase 3 study, the same MenABCWY 0- to 6-month schedule induced bactericidal immune responses against a panel of 110 MenB strains [27]. Using the endogenous complement hSBA assay [32], analysis of samples with bactericidal activity against the tested MenB strains showed 78% breadth of immune response, while 84% of participants had sera that killed ≥70% of tested strains [27]. The 110 MenB strain panel is epidemiologically relevant, representing approximately 95% of invasive MenB disease isolates in the US and 89% of global isolates [33].

The MenABCWY vaccine was well tolerated in MenACWY-primed individuals with no safety concerns identified. Other studies [19, 20, 25], including the previous phase 3 study [31], showed higher rates of injection site reactions in groups that received MenB and MenB-containing vaccines than in MenACWY groups, and this was also observed in the present study. As expected, reactogenicity was comparable between groups following the second injection since 4CMenB was administered to the MenACWY group at month 6. Injection site pain, fatigue, and headache were the most commonly reported solicited events in both groups. Rates of unsolicited AEs were similar between groups, and no increase in AE reporting was observed after the second dose of MenABCWY. Overall, MenABCWY had a clinically acceptable reactogenicity and safety profile in adolescents and young adults that was generally consistent with that of 4CMenB, as previously observed [19, 20, 25, 31].

This study has several limitations. The lack of broad racial diversity in the study population could reduce the generalizability of the results to different groups, and antibody persistence was not evaluated beyond 1 month postvaccination. Also, the results may not apply to other pentavalent MenB-containing vaccines or MenACWY vaccines. A strength of this study is the consistency with results from previous phase 2 and 3 studies of this MenABCWY vaccine [19–27, 31], which is a combination of licensed 4CMenB and MenACWY-CRM vaccines with well-established safety and effectiveness profiles [7].

In summary, immune responses against serogroups A, C, W, and Y following 1 and 2 doses of investigational MenABCWY vaccine are noninferior to those following a single MenACWY-CRM dose in healthy adolescents and young adults who had received a MenACWY vaccine dose previously. After 2 MenABCWY doses administered 6 months apart, robust immune responses were observed against serogroups A, C, W, and Y, and against the 4 MenB indicator strains. The MenABCWY vaccine was well tolerated, with a favorable reactogenicity and safety profile. These findings provide further supporting evidence for the development of the MenABCWY vaccine for broad protection against IMD.

## Supplementary Material

ciae622_Supplementary_Data

## Supplementary Data

Supplementary materials are available at *Clinical Infectious Diseases* online. Consisting of data provided by the authors to benefit the reader, the posted materials are not copyedited and are the sole responsibility of the authors, so questions or comments should be addressed to the corresponding author.

## Notes

***Author contributions.*** All authors participated in study design, implementation, or analysis; the interpretation of the study; and the development of this manuscript. All authors had full access to the data and gave final approval before submission.

***Acknowledgments.*** The authors thank the participants, site personnel, and investigators who took part in the study. The authors thank Enovalife Medical Communication Service Center for editorial assistance and manuscript coordination, on behalf of GSK. Joanne Knowles (independent medical writer, on behalf of GSK) provided medical writing support.

***Trademarks.*** Bexsero and Menveo are trademarks owned by or licensed to GSK. Penbraya and Trumenba are trademarks of Pfizer.

***Data availability.*** Please refer to the following web link to access GSK's data sharing policies and, as applicable, seek anonymized subject-level data (https://www.gsk-studyregister.com/en/).

***Disclaimer.*** GSK was involved in all stages of study conduct, including analysis of the data. GSK also took in charge all costs associated with the development and publication of this manuscript.

***Financial support***. This work was supported by GSK.
