# Supplementary material for: Immunogenicity, Reactogenicity, and Safety of a Pentavalent Meningococcal ABCWY Vaccine in Adolescents and Young Adults Who Had Previously Received a Meningococcal ACWY Vaccine: A Phase 3, Randomized Controlled Clinical Study
Source: Clin Infect Dis. 2024 Dec 26;80(4):752–60. doi: 10.1093/cid/ciae622 (PMC12043065; doi:10.1093/cid/ciae622)
Supplement: ciae622_Supplementary_Data [file ciae622_supplementary_data.docx]

# SUPPLEMENTARY MATERIAL

## BOOST Study Group

Investigators: Adebayo Akinsola, Madhavi Ampajwala, Mark Arya, Andrew Bartlett, Divya Batra, Mark Theo Bloch, Jose Bordon, William Byars, Jeremy Peter James Carr, Ana Ceballos, Ferdinandus de Looze, Mercedes Deluca, Joseph Domachowske, Rand Farjo, Ezekiel Fink, Angela Gentile, Jennifer Gilsoul, Elizabeth Anne Gunner, Anil K Gupta, Alejandro Hoberman, Matthew Hong, Julie Kasarjian, Conrado Juan Llapur, Darvy Mann, Gonzalo Perez Marc, Paul G Matherne, Gretchen Mitchell, Terry Nolan, Mora Nair Obed, John O’Mahony, Peter Richmond, Fernando Oscar Riera, Walter Rok, Louis Saravolatz, Peter Silas, Adriana Elvira Soto, Katherine Sullivan, Joseph Surber, Ricardo Augusto Teijeiro, Florence Tiong, Olga Voloshyna, Ushma Wadia, Garry Wallace, Clifford Yut

GSK: Chiranjiwi Bhusal, Maria Lattanzi, Isabelle Lechevin, Danielle Morelle, Thembile Mzolo, Stefanie Raulier, Daniela Toneatto, Mauro Trapani

## Study Endpoints

*Primary endpoints*

- Percentages of participants with a 4-fold rise in hSBA titers against *Neisseria meningitidis* serogroups A, C, W, and Y at 1 month after the second vaccine dose for the MenABCWY group and 1 month after the single MenACWY-CRM vaccine dose for the MenACWY group relative to baseline.
- Percentages of participants with a 4-fold rise in hSBA titers against *N. meningitidis* serogroups A, C, W, and Y at 1 month after the first vaccine dose for the MenABCWY group and 1 month after the single MenACWY-CRM vaccine dose for the MenACWY group relative to baseline.
- Frequencies and percentages of participants with solicited administration site events (i.e., injection site pain, erythema, swelling, induration) and solicited systemic events (i.e., fever, nausea, fatigue, myalgia, arthralgia, headache) during the 7 days (including the day of vaccination) following vaccination at Month 0 (for the MenABCWY and MenACWY groups) and Month 6 (for the MenABCWY group).
- Frequencies and percentages of participants with any unsolicited adverse events (AEs) (including all serious AEs [SAEs], AEs leading to withdrawal, AEs of special interest [AESIs], and medically attended AEs) during the 30 days (including the day of vaccination) following vaccination at Month 0 (for the MenABCWY and MenACWY groups) and Month 6 (for the MenABCWY group).
- Percentages of participants with SAEs, AEs leading to withdrawal, AESIs, and medically attended AEs throughout the study period (Month 0 to Month 12).

*Secondary endpoints*

- Percentages of participants with hSBA titers greater than or equal to the lower limit of quantitation (≥LLOQ) against serogroups A, C, W, and Y at baseline and at 1 month after the first and second vaccination for the MenABCWY group, and at baseline and 1 month after the single MenACWY-CRM vaccine dose for the MenACWY group.
- Antibody geometric mean titers (GMTs) against serogroups A, C, W, and Y at baseline and at 1 month after the first and second vaccinations for the MenABCWY group, and at baseline and 1 month after the single MenACWY-CRM vaccine dose for the MenACWY group.
- Antibody geometric mean ratios (GMRs) against serogroups A, C, W, and Y at 1 month after the first and second vaccinations for the MenABCWY group as compared to baseline and at 1 month after the single MenACWY-CRM vaccine dose for the MenACWY group as compared to baseline.
- Percentages of participants with hSBA titers ≥LLOQ for each and all *N. meningitidis* serogroup B indicator strains at baseline and at 1 month after the second vaccination for the MenABCWY group.
- Percentages of participants with a 4-fold rise in hSBA titers against each *N. meningitidis* serogroup B indicator strain at 1 month after the second vaccine dose relative to baseline for the MenABCWY group.
- GMTs against each serogroup B indicator strain at baseline and at 1 month after the second vaccine dose for the MenABCWY group.
- GMRs against each serogroup B indicator strain at 1 month after the second vaccine dose as compared to the baseline for the MenABCWY group.

## Lower Limits of Quantitation

The lower limit of quantitation was 12, 8, 8, and 10 for serogroups A, C, W, and Y, respectively, and 5, 15, 4, and 6 for *N. meningitidis* serogroup B indicator strains fHbp, NadA, NHBA, and PorA, respectively.

## Additional Information on Statistical Analysis

The sample size and power calculations were done using the software PASS 12 (www.ncss.com) and a 1-sided score test (Miettinen O, Nurminen M. Stat Med. **1985**;4:213–26) was used.

The first primary immunogenicity objective was to demonstrate the noninferiority of the antibody response to MenABCWY vaccine compared to MenACWY vaccine given to healthy participants previously primed with MenACWY at 1 month after the second MenABCWY vaccination and 1 month after the MenACWY vaccination. This translated to the following hypotheses:

H_0_: (p_MenABCWY_(i)_ - p_MenACWY_(i)_) ≤ −10%

versus

H_1_: (p_MenABCWY_(i)_ - p_MenACWY_(i)_) > −10%

Where p_MenABCWY_(i)_ denotes the percentages of participants with 4-fold rise in hSBA titers for serogroups i=A, C, W and Y, 1 month after the second vaccination in the MenABCWY group and p_MenACWY_(i)_ denotes the percentages of participants with 4-fold rise in hSBA titers for serogroups i=A, C, W and Y, 1 month after the single vaccination in the MenACWY group.

The same formula was used for the second primary immunogenicity objective (to demonstrate the noninferiority of the antibody response to MenABCWY vaccine compared to MenACWY vaccine given to healthy MenACWY-primed participants at 1 month after the first MenABCWY vaccination and 1 month after the MenACWY vaccination), substituting ‘1 month after the second vaccination in the MenABCWY group’ with ‘1 month after the first vaccination in the MenABCWY group’.

## Vaccine Preparation and Injection

The MenABCWY vaccine was prepared immediately before injection by reconstituting the lyophilized powder that had the same composition as MenACWY-CRM [1] with a liquid suspension containing the MenB component with the same composition as 4CMenB [2]. MenACWY-CRM was prepared immediately before injection by reconstituting the MenA lyophilized powder with a MenCWY liquid suspension. Vaccine (0.5 mL) injections were administered intramuscularly into the deltoid region, preferably of the nondominant arm.

***References***

1. Cooper B, DeTora L, Stoddard J. Menveo: a novel quadrivalent meningococcal CRM_197_ conjugate vaccine against serogroups A, C, W-135 and Y. Expert Rev Vaccines **2011**; 10:21–33.

2. O’Ryan M, Stoddard J, Toneatto D, Wassil J, Dull PM. A multi-component meningococcal serogroup B vaccine (4CMenB): the clinical development program. Drugs **2014**; 74:15–30.

# **Supplementary Table 1. Human Serum Bactericidal Antibody Geometric Mean Titers (GMTs)** **Against Meningococcal Serogroups A, C, W, and Y at Baseline and 1 Month After 1 (Month 1) and 2 (Month 7) MenABCWY Doses and 1 Month (Month 1) After the MenACWY-CRM Dose, and GMT Ratios (Full Analysis Set)**

| Serogroup  Timepoint | MenABCWY group | | MenACWY group | |
| --- | --- | --- | --- | --- |
|  | No. of Participants | hSBA GMT (95% CI) | No. of Participants | hSBA GMT (95% CI) |
| Serogroup A |  |  |  |  |
| Baseline | 546 | 15.3 (13.1, 17.9) | 549 | 16.3 (14.0, 19.1) |
| Month 1 | 605 | 670.8 (594.7, 756.6) | 585 | 1282.6 (1135.8, 1448.2) |
| Month 7 | 213 | 645.2 (544.1, 765.2) | … | … |
| Month 1/Baseline | 539 | 44.0 (36.9, 52.4) | 539 | 76.9 (64.5, 91.7) |
| Month 7/Baseline | 196 | 44.7 (34.7, 57.7) | … | … |
| Serogroup C |  |  |  |  |
| Baseline | 601 | 31.9 (26.6, 38.2) | 584 | 29.8 (24.8, 35.7) |
| Month 1 | 609 | 2945.7 (2471.1, 3511.4) | 593 | 2552.3 (2138.7, 3045.9) |
| Month 7 | 211 | 2350.1 (1809.7, 3052.0) | … | … |
| Month 1/Baseline | 597 | 92.9 (77.1, 111.8) | 583 | 86.4 (71.6, 104.1) |
| Month 7/Baseline | 205 | 68.3 (52.2, 89.5) | … | … |
| Serogroup W |  |  |  |  |
| Baseline | 597 | 12.1 (10.3, 14.2) | 583 | 11.1 (9.5, 13.0) |
| Month 1 | 607 | 1899.6 (1638.7, 2202.0) | 592 | 1665.6 (1435.6, 1932.6) |
| Month 7 | 212 | 1173.6 (940.3, 1464.9) | … | … |
| Month 1/Baseline | 591 | 154.1 (125.8, 188.7) | 581 | 150.8 (123.0, 185.0) |
| Month 7/Baseline | 207 | 97.7 (72.5, 131.5) | … | … |
| Serogroup Y |  |  |  |  |
| Baseline | 600 | 12.84 (11.2, 14.8) | 576 | 11.9 (10.3, 13.7) |
| Month 1 | 606 | 1590.7 (1380.8, 1832.6) | 591 | 1578.4 (1369.0, 1819.9) |
| Month 7 | 210 | 1130.5 (916.9, 1394.0) | … | … |
| Month 1/Baseline | 593 | 124.1 (103.2, 149.2) | 573 | 134.7 (111.8, 162.3) |
| Month 7/Baseline | 202 | 82.5 (62.8, 108.2) | … | … |

Abbreviations: CI, confidence interval; hSBA, human serum bactericidal antibody; GMT, geometric mean titer; MenABCWY group, received 2 doses of meningococcal serogroups ABCWY vaccine at study months 0, 6; MenACWY group, received meningococcal serogroups ACWY CRM_197_-glycoconjugate vaccine at month 0.

# **Supplementary Table 2. Human Serum Bactericidal Antibody Geometric Mean Titers (GMTs) in the MenABCWY Group Against Each Meningococcal Serogroup B Indicator Strain and GMT Ratios (Full Analysis Set)**

| MenB indicator strain  Timepoint | No. of Participants | hSBA GMT (95% CI) |
| --- | --- | --- |
| fHbp |  |  |
| Baseline | 184 | 2.8 (2.5, 3.2) |
| Month 1 | 183 | 4.6 (3.6, 5.8) |
| Month 7 | 165 | 17.6 (13.5, 22.8) |
| Month 7/Baseline | 163 | 6.3 (4.8, 8.2) |
| NadA |  |  |
| Baseline | 183 | 8.6 (7.4, 10.1) |
| Month 1 | 184 | 22.6 (16.3, 31.4) |
| Month 7 | 165 | 143.6 (106.7, 193.3) |
| Month 7/Baseline | 162 | 16.7 (12.3, 22.7) |
| NHBA |  |  |
| Baseline | 183 | 3.3 (2.6, 4.2) |
| Month 1 | 184 | 7.7 (5.4, 11.0) |
| Month 7 | 164 | 24.8 (19.2, 32.1) |
| Month 7/Baseline | 161 | 7.7 (6.1, 9.7) |
| PorA |  |  |
| Baseline | 184 | 3.1 (2.9, 3.4) |
| Month 1 | 183 | 4.9 (3.7, 6.3) |
| Month 7 | 164 | 11.4 (8.6, 15.2) |
| Month 7/Baseline | 162 | 3.7 (2.8, 4.8) |

Abbreviations: CI, confidence interval; fHbp, factor H binding protein; hSBA, human serum bactericidal antibody; GMT, geometric mean titer; MenABCWY group, received 2 doses of meningococcal serogroups ABCWY vaccine at study months 0, 6; MenB, meningococcal serogroup B; NadA, *Neisseria* adhesin A; NHBA, neisserial heparin-binding antigen; PorA, Porin A.

# **Supplementary Table 3. Summary of Numbers and Percentages of Participants Reporting Solicited Administration Site and Systemic Adverse Events Within 7 Days of Each Injection (Solicited Safety Set)**

|  | MenABCWY group, n (%; 95% CI) | MenACWY group, n (%; 95% CI) |
| --- | --- | --- |
| Injection 1 | N=626 | N=621 |
| Any AE | 529 (84.5; 81.4–87.3) | 372 (59.9; 55.9–63.8) |
| Administration site AE | 488 (78.0; 74.5–81.1) | 197 (31.7; 28.1–35.5) |
| Systemic AE | 369 (58.9; 55.0–62.8) | 320 (51.5; 47.5–55.5) |
| Injection 2 | N=571 | N=562 |
| Any AE | 405 (70.9; 67.0–74.6) | 431 (76.7; 73.0–80.1) |
| Administration site AE | 378 (66.2; 62.2–70.1) | 402 (71.5; 67.6–75.2) |
| Systemic AE | 253 (44.3; 40.2–48.5) | 266 (47.3; 43.1–51.6) |
| Injections 1 and 2 | N=626 | N=621 |
| Any AE | 556 (88.8; 86.1–91.2) | 522 (84.1; 80.9–86.9) |
| Administration site AE | 521 (83.2; 80.1–86.1) | 447 (72.0; 68.3–75.5) |
| Systemic AE | 427 (68.2; 64.4–71.9) | 404 (65.1; 61.2–68.8) |

Abbreviations: AE, adverse event; CI, confidence interval; MenABCWY group, received meningococcal serogroups ABCWY vaccine at each injection; MenACWY group, received meningococcal serogroups ACWY CRM_197_-glycoconjugate vaccine at injection 1, 4CMenB at injection 2. N, number of participants in group who provided solicited AE data; n, number of participants in solicited AE category.

# **Supplementary Table 4. Numbers and Percentages of Participants Reporting Unsolicited Adverse Events Within 30 Days of Receiving a Vaccine Dose and Throughout the Study Period (Unsolicited Safety Set)**

|  | MenABCWY group, n (%)  N=626 | MenACWY group, n (%)  N=621 |
| --- | --- | --- |
| Within 30 days of any vaccination | | |
| Unsolicited AEs | 150 (24.0) | 150 (24.2) |
| Related unsolicited AEs | 18 (2.9) | 31 (5.0) |
| Serious AEs | 4 (0.6) | 1 (0.2) |
| Related serious AEs | 0 | 0 |
| Medically attended unsolicited AEs | 82 (13.1) | 77 (12.4) |
| Unsolicited AE leading to withdrawal | 2 (0.3) | 3 (0.5) |
| Unsolicited AESI | 0 | 1 (0.2) |
| Deaths | 0 | 0 |
| Throughout study period | | |
| Unsolicited AEs | 266 (42.5) | 264 (42.5) |
| Related unsolicited AEs | 21 (3.4) | 32 (5.2) |
| Serious AEs | 18 (2.9) | 7 (1.1) |
| Related serious AEs | 0 | 0 |
| Medically attended unsolicited AEs | 223 (35.6) | 206 (33.2) |
| Unsolicited AE leading to withdrawal | 4 (0.6) | 6 (1.0) |
| Unsolicited AESI | 0 | 4 (0.6) |
| Deaths | 1 (0.2) | 1 (0.2) |

Abbreviations: AE, adverse event; AESI, adverse event of special interest; MenABCWY group, received 2 doses of meningococcal serogroups ABCWY vaccine at study months 0, 6; MenACWY group, received meningococcal serogroups ACWY CRM_197_-glycoconjugate vaccine at month 0; N, number of participants; n, number of participants in unsolicited AE category.
